# Supplementary material for: Health outcomes, healthcare use and development in children born into or growing up in single-parent households: a systematic review study protocol
Source: BMJ Open. 2021 Feb 11;11(2):e043361. doi: 10.1136/bmjopen-2020-043361 (PMC7880085; doi:10.1136/bmjopen-2020-043361)
Supplement: Supplementary data [file bmjopen-2020-043361supp001.pdf]

APPENDIX 1 – SEARCH STRATEGY

	**PUBMED**	
#1	single parents	"single parent"[MeSH Terms] OR "single parent*"[Title/Abstract] OR "lone parent*"[Title/Abstract] OR "lone mother*"[Title/Abstract] OR "lone father*"[Title/Abstract] OR "single father*"[Title/Abstract] OR "single mother*"[Title/Abstract] OR "single parent family"[Title/Abstract] OR "single parent families"[Title/Abstract] OR "single-parent family"[MeSH Terms] OR "unmarried parent*"[Title/Abstract] OR "marital status"[Title/Abstract] OR "marital status"[MeSH Terms] OR "family structure"[Title/Abstract]
#2	birth outcomes	"birth outcome*"[Title/Abstract] OR "birth weight"[MeSH Terms] OR "birth weight"[Title/Abstract] OR "infant, low birth weight"[MeSH Terms] OR "infant, premature"[MeSH Terms] OR "gestational age"[Title/Abstract] OR "preterm birth"[Title/Abstract] OR "congenital anomal*"[Title/Abstract]
#3	mortality	"child mortality"[Title/Abstract] OR "infant mortality"[Title/Abstract] OR "perinatal mortality"[Title/Abstract] OR "fetal mortality"[Title/Abstract] OR "stillbirth"[Title/Abstract] OR "stillbirth"[MeSH Terms]
#4	physical health	"child health"[MeSH Terms] OR "child health"[Title/Abstract] OR "pediatric obesity"[MeSH Terms] OR "child nutrition"[Title/Abstract] OR "eating habits"[Title/Abstract] OR "oral health"[Title/Abstract] OR "motor skills"[Title/Abstract]
#5	mental health and development	mental health[MeSH Terms] OR "educational status"[MeSH Terms] OR "cognitive abilit*"[Title/Abstract] OR "educational attainment"[Title/Abstract] OR "educational status"[Title/Abstract] OR "child behavior disorders"[MeSH Terms] OR "depressive disorder"[MeSH Terms] OR "anxiety disorders"[MeSH Terms]
#6	healthcare use	"hospital admission"[Title/Abstract] OR "emergency admission"[Title/Abstract] OR "healthcare use"[Title/Abstract] OR "health care use"[Title/Abstract] OR "healthcare utilisation"[Title/Abstract] OR "health visitor"[Title/Abstract] OR "primary care"[Title/Abstract] OR "vaccination"[MeSH Terms]
#7		#2 OR #3 OR #4 OR #5 OR #6
#8		#1 AND #7
#9		limit #8 to 2000-2020

	**SCOPUS**	
#1	single parents	TITLE-ABS ("single parent*" OR "lone parent*" OR "lone mother*" OR "lone father*" OR "single father*" OR "single mother*" OR "single parent family" OR "single parent families" OR "unmarried parent*" OR "marital status" OR "family structure")
#2	birth outcomes	TITLE-ABS ("birth outcome*" OR "birthweight" OR "gestational age" OR "preterm birth" OR "congenital anomal*")
#3	mortality	TITLE-ABS ("child mortality" OR "infant mortality" OR "perinatal mortality" OR "fetal mortality" OR "stillbirth")

#4	physical health	TITLE-ABS ("child health" OR "oral health" OR "pediatric obesity" OR "child nutrition" OR "eating habits" OR "motor skills")
#5	mental health and development	TITLE-ABS ("mental health" OR "disruptive behavior" OR "disruptive behaviour" OR "cognitive abilit*" OR "educational attainment" OR "educational status" OR "child development")
#6	healthcare use	TITLE-ABS ("healthcare use" OR "hospitalization" OR "hospitalisation" OR "hospital admission" OR "emergency admission" OR "vaccinat*")
#7		#2 OR #3 OR #4 OR #5 OR #6
#8		#1 AND #7
#9		limit #8 to 2000-2020

	**SCOPUS**	
#1	single parents	ti("single parent?" OR "lone parent?" OR "lone mother?" OR "lone father?" OR "single father?" OR "single mother?" OR "single parent family" OR "single parent families" OR "unmarried parent?" OR "marital status" OR "family structure") OR ab("single parent?" OR "lone parent?" OR "lone mother?" OR "lone father?" OR "single father?" OR "single mother?" OR "single parent family" OR "single parent families" OR "unmarried parent?" OR "marital status" OR "family structure")
#2	birth outcomes	ti("birth outcome?" OR "birthweight" OR "birth weight" OR "gestational age" OR "preterm birth" OR "congenital anomal?") OR ab("birth outcome?" OR "birthweight" OR "birth weight" OR "gestational age" OR "preterm birth" OR "congenital anomal?")
#3	mortality	ti("child mortality" OR "infant mortality" OR "perinatal mortality" OR "fetal mortality" OR "stillbirth") OR ab("child mortality" OR "infant mortality" OR "perinatal mortality" OR "fetal mortality" OR "stillbirth")
#4	physical health	ti("child health" OR "oral health" OR "pediatric obesity" OR "child nutrition" OR "eating habits" OR "motor skills") OR ab("child health" OR "oral health" OR "pediatric obesity" OR "child nutrition" OR "eating habits" OR "motor skills")
#5	mental health and development	ti("mental health" OR "disruptive behavior" OR "disruptive behaviour" OR "cognitive abilit?" OR "educational attainment" OR "educational status" OR "child development") OR ab("mental health" OR "disruptive behavior" OR "disruptive behaviour" OR "cognitive abilit?" OR "educational attainment" OR "educational status" OR "child development")
#6	healthcare use	ti("hospitalisation" OR "hospitalization" OR "hospital admission" OR "emergency admission" OR "healthcare use" OR "vaccinat?") OR ab("hospitalisation" OR "hospitalization" OR "hospital admission" OR "emergency admission" OR "healthcare use" OR "vaccinat?")
#7		#2 OR #3 OR #4 OR #5 OR #6
#8		#1 AND #7
#9		limit #8 to 2000-2020
