# Supplementary material for: Health outcomes, healthcare use and development in children born into or growing up in single-parent households: a systematic review study protocol
Source: BMJ Open. 2021 Feb 11;11(2):e043361. doi: 10.1136/bmjopen-2020-043361 (PMC7880085; doi:10.1136/bmjopen-2020-043361)
Supplement: Author's manuscript [file bmjopen-2020-043361.draft_revisions.pdf]

BMJ Open is committed to open peer review. As part of this commitment we make the peer review history of every article we publish publicly available.

When an article is published we post the peer reviewers' comments and the authors' responses online. We also post the versions of the paper that were used during peer review. These are the versions that the peer review comments apply to.

The versions of the paper that follow are the versions that were submitted during the peer review process. They are not the versions of record or the final published versions. They should not be cited or distributed as the published version of this manuscript.

BMJ Open is an open access journal and the full, final, typeset and author-corrected version of record of the manuscript is available on our site with no access controls, subscription charges or pay-per-view fees (http://bmjopen.bmj.com).

If you have any questions on BMJ Open's open peer review process please email info.bmjopen@bmj.com

BMJ Open

Health outcomes and healthcare use in children born into or growing up in single parent households: A systematic review

Journal:	*BMJ Open*
Manuscript ID	bmjopen-2020-043361
Article Type:	Protocol
Date Submitted by the Author:	03-Aug-2020
Complete List of Authors:	Lut, Irina; UCL, GOS Institute of Child Health<br>Woodman, Jenny; UCL, Institute of Education<br>Armitage, Alice; UCL, GOS Institute of Child Health<br>Ingram, Elizabeth; UCL, Department of Applied Health Research<br>Hardelid, Pia; UCL, GOS Institute of Child Health
Keywords:	PAEDIATRICS, PUBLIC HEALTH, Community child health < PAEDIATRICS

SCHOLARONE™
Manuscripts

BMJ

I, the Submitting Author has the right to grant and does grant on behalf of all authors of the Work (as defined in the below author licence), an exclusive licence and/or a non-exclusive licence for contributions from authors who are: i) UK Crown employees; ii) where BMJ has agreed a CC-BY licence shall apply, and/or iii) in accordance with the terms applicable for US Federal Government officers or employees acting as part of their official duties; on a worldwide, perpetual, irrevocable, royalty-free basis to BMJ Publishing Group Ltd ("BMJ") its licensees and where the relevant Journal is co-owned by BMJ to the co-owners of the Journal, to publish the Work in this journal and any other BMJ products and to exploit all rights, as set out in our licence.

The Submitting Author accepts and understands that any supply made under these terms is made by BMJ to the Submitting Author unless you are acting as an employee on behalf of your employer or a postgraduate student of an affiliated institution which is paying any applicable article publishing charge ("APC") for Open Access articles. Where the Submitting Author wishes to make the Work available on an Open Access basis (and intends to pay the relevant APC), the terms of reuse of such Open Access shall be governed by a Creative Commons licence – details of these licences and which Creative Commons licence will apply to this Work are set out in our licence referred to above.

Other than as permitted in any relevant BMJ Author's Self Archiving Policies, I confirm this Work has not been accepted for publication elsewhere, is not being considered for publication elsewhere and does not duplicate material already published. I confirm all authors consent to publication of this Work and authorise the granting of this licence.

For peer review only

Title

Health outcomes and healthcare use in children born into or growing up in single parent households: A systematic review

Authors
Irina Lut[1], Jenny Woodman[2], Alice Armitage[1], Elizabeth Ingram[3], Pia Hardelid[1]

1. UCL GOS Institute of Child Health
2. UCL Institute of Education
3. UCL Department of Applied Health Research

Email addresses: i.lut@ucl.ac.uk ; j.woodman@ucl.ac.uk ; alice.armitage.13@ucl.ac.uk ; e.ingram.17@ucl.ac.uk ; p.hardelid@ucl.ac.uk

Corresponding author:
Irina Lut
Population, Policy and Practice Research and Teaching Department
UCL GOS Institute of Child Health
30 Guildford St, London WC1N 1EH
Tel: 07960087974
Email: i.lut@ucl.ac.uk

Tables: 0
Figures: 0
Appendices: 1
Word Count: 2225

For peer review only

Abstract

Introduction

Up to a quarter of all children globally live in single parent households. Studies have concluded that children who grow up with continuously married parents have better health outcomes than children who grow up with single or separated parents. This is consistent across key domains including physical health, psychological wellbeing and educational attainment. Possible explanations include higher poverty and time limitations of parental engagement within single parent families, but these only represent a narrow range of mechanisms. We aim to identify and synthesise the evidence on how being born into and/or living in a single parent household as a child impacts on health, development and healthcare use compared to living in a two-parent household, and factors that may be driving differences.

Methods and Analysis

We will search Pubmed, Scopus, and ERIC and adapt our search terms for search engines and grey literature sites to include relevant conference abstracts and grey literature. We will restrict results to English language publications from 2000-2020 and screen against inclusion criteria. We will categorise main outcomes into five groups of outcomes: birth outcomes, mortality, physical health, mental health, development, and healthcare use. We will use the Newcastle-Ottawa scale to assess the methodological quality of studies. Narrative synthesis will form the primary analysis in the review. Synthesis of effect estimates without meta-analysis will follow the Synthesis Without Meta-Analysis (SWiM) guidelines.

Ethics and Dissemination

All documents used are publicly accessible. We will submit results to a peer-reviewed journal and international social science conferences. We will communicate results with single parent groups and relevant charitable organisations. This review will also be included in IL's thesis.

Registration Details

The protocol has been accepted to the International Prospective Register for Systematic Reviews (PROSPERO) database, registration number CRD42020197890.

For peer review only

Article Summary

Strengths and limitations of this study:

- This review will fill an evidence gap on the drivers and protective factors that influence the health and development of children in single parent households
- A robust methodology and extensive search strategy will support clear results to inform policies and interventions to support single household families

- Findings from included studies will likely be heterogenous in terms of definitions of single households and definitions and measurements of outcomes, precluding meta-analysis and making subgroup analyses difficult

Introduction

Between 10% and 25% of children in member countries of the Organisation for Economic Cooperation and Development (OECD) live in single parent households (1). In Great Britain, 15% of all families are headed by a single parent (2). The proportion of single parent families in Great Britain has remained stable over the last 20 years (3) following an increase of single parent families between 1970 and 1995 (4). The key reasons for this increase were rising levels of divorce and partnership breakdown during the 1970s and 1980s (2, 5) and an increase in the number of births to single women since the mid-1980s (4, 6, 7).

Multiple studies have concluded that children who grow up with continuously married parents have better outcomes than children who grow up with single parents or children whose parents separate during childhood (8-10). This is consistent across key domains including physical health (11), psychological wellbeing (12) and educational attainment (13).

A systematic review of maternal marital status and birth outcomes from 2010 has summarised the current literature on risks of an infant being born with low birth weight (<2500 g), preterm birth (<37 weeks gestational age) or small for gestational age (below the 10th percentile for babies of the same gestational age) among married and unmarried women (14). Findings identify significantly increased odds of low birth weight, preterm birth and small for gestational age births among unmarried women compared to married women. A further systematic review found that children in single parent households have higher body mass index and obesogenic behaviours such as insufficient physical activity and increased television viewing time compared to children living with two parents (15).

Children of single parents are at higher risk of living in poverty and deprivation compared to children growing up in coupled families (16). In 2018, 49% of children in single parent families live in poverty in England compared to 25% of children in coupled families (17). Socio-economic factors such as income, occupation and education (also referred to as socio-economic status or SES), are strongly associated with parental and child wellbeing (18). While it is clear that single parent families are disadvantaged with respect to socioeconomic status and health outcomes, it remains unclear whether SES fully explains differences in outcomes for children of single mothers compared to children with two parents. This is difficult to examine since the likelihood of becoming a single mother is very strongly associated with SES. Women with lower SES (based on their father's occupation) were up to six times more likely to become single mothers in a study of three large British suveys (19). The rate of relationship breakdown resulting in single motherhood was found to be almost double among women in unskilled work compared to women in professional or managerial roles (19).

A substantial body of research also exists on the health impact of parental relationship breakdown (a mode of entry into single parenthood) or reforming, on child health (20).

For peer review only

However, a review of literature up to 2005 concludes that children with two continuously married parents attain better cognitive and emotional outcomes compared to children with only one biological parent in the household (9) and this is more plausibly explained by higher deprivation and lower education among single mothers than by the impacts of a parental relationship breakdown (4).

Research on single parenthood has focused largely on single mothers, who head approximately 88% of single parent families globally (21). Women more often parent alone due to breakdown of a relationship or pursue motherhood without a partner from the point of conception than men (4). Research on the quality and quantity of fathering exists but has tended to focus on the impact of father absence rather than single father families (22). There are well-described challenges to capturing fathers in research (23, 24); even less is known about different types of single fathers than about about different types of single mothers.

Historically, official statistics have relied heavily on marital status to define single motherhood. Women who have had children with cohabiting partners and lived in a two-parent household have previously been grouped with single mothers, leading to inflation of the number of families that appeared to be led by non-partnered women (4). This is despite cohabiting households with children being one of the fastest growing family forms between 1980s-2000s in the UK and other countries (3). Single mothers who have separated from a partner, either via divorce or relationship breakdown, are likely to be different from women with no partner who become mothers (4) and report different parenting experiences (25). Capturing nuances in single parent households may be critical in understanding why children of single mothers have poorer outcomes compared to children of coupled mothers and identifying protective factors. Distinguishing between different types of and all routes into single parenthood is important as family structures have become more complex and new non-traditional family forms are being recognised (4, 26). Definitions and terminology matter not only to make sure we understand the comparisons we are making between groups but also to ensure that negative or stigmatising narratives associated with single motherhood are not perpetuated (27).

In this systematic review we will compare a range of health and development outcomes among children living in single parent households and children living in coupled parent households, and identify factors that may be driving differences. We will focus particularly on children who remain in single parent versus continuously coupled families but also include comparisons with cohabiting and married coupled families or different types of single parent families where available. We aim to fill gaps in evidence by exploring whether health disparities between children of single parents and children of coupled parents persist after adjustment for socioeconomic characteristics, presenting findings that explain the differences and reporting protective factors that allow children to be healthy in a single parent family. Our findings will highlight areas where policy change or public health interventions might help improve health of the large numbers of children living in single parent households.

For peer review only

Methods and Analysis

Aims and research questions

The aim of this review is to systematically identify and synthesise the evidence on how being born into and/or living in a single parent household as a child impacts on health, development and healthcare use compared to living in a two-parent household, and factors that may be driving differences.

This systematic review will answer the following questions:

1. How do health and development outcomes compare among children and young people (less than 18 years old) growing up in single parent and coupled parent households?
2. What factors influence any observed differences in child health and development outcomes between children of single parents versus coupled parents?

Searches

We will search for the concepts 'single parents' AND 'child health' OR 'child development' outcomes using indexed medical subject headings (MeSH) and free text terms, restricting results to English language publications from 2000-2020. We will search three databases which index medical, social science and education research: Pubmed, Scopus, and ERIC. We will identify additional relevant results through backwards and forwards citation searching and grey literature search engines. We provide the full list of search concepts and terms in appendix 1 (carried out on 15.07.20). We will adapt our search terms for search engines like Google Scholar and Scirus, and refer to the Canadian Agency for Drugs and Technologies in Health (CADTH) guidance for links to grey literature sites relevant in the UK context, to search for conference abstracts and grey literature or additional peer-reviewed articles.

Two researchers (IL and AA or IL and EI) will independently screen all results at the title and abstract stage and further screen full texts if the study's eligibility for inclusion remains unclear from the first screen. A third reviewer (PH or JW) will resolve any discrepancies.

Inclusion and exclusion criteria

The population of interest is children who have experienced living in a single parent household at any time during childhood (aged less than 18 years) and have at least one of the health outcomes measured in the study before the age of 18 years. We will include studies if the single parent is living with dependent children and does not have a partner living in the same home. We will exclude studies that focus exclusively on the health effects of parental relationship breakdown and do not also investigate the effects of single parenthood. We will include studies with any definition and measure of the five types of outcomes.

Outcomes

We present the main outcomes in this review in five groups:

1. <u>Birth outcomes</u>: including birth weight, low birth weight (<2500g), very low birth weight (<1500g), gestational age, small for gestational age (<10th percentile), preterm birth, congenital anomalies

For peer review only - http://bmjopen.bmj.com/site/about/guidelines.xhtml

2. Mortality outcomes: including stillbirth, perinatal mortality, child mortality
3. Physical health outcomes: including nutrition, weight, oral health, motor skills
4. Mental health and development outcomes: including disruptive behaviour, anxiety or depressive disorders, autism-spectrum disorders, psychosis, cognitive abilities (problem solving, memory, language/communication, early years educational attainment), social-emotional development (personal-social skills)
5. Healthcare use outcomes: including any hospital admission (planned or emergency), vaccinations, visits to primary care, contact with health visitors or well-child checks

Data extraction and management

For each included study, we will extract information on study authors and date of publication, study setting and period, study design (including selection criteria, number of participants and analysis), timings of single parenthood and outcomes of the study. All management of records and data will be done within the EPPI-Reviewer software.

Studies will be grouped by exposure groups and main outcomes. Exposure groups are likely to differ by the type, timing and duration of parental relationship status. Authors will be contacted if the time parameters of single parenthood is not clear from the published work. Outcomes will be grouped into the four main outcome groups defined above. We will use the Newcastle-Ottawa scale to assess the methodological quality of studies (28). We will use the Risk of Bias in Systematic Reviews (ROBIS) tool to guide our methods, originally designed for assessing risk of bias in the systematic review process of published reviews (29).

Synthesis and meta-analysis

Given the range of outcomes and the likely diversity in the way single parent households are defined across studies, we expect that the included studies will be too heterogeneous to carry out meta-analyses. Narrative synthesis will therefore form the primary analysis in the review. We will incorporate the four main elements described in the Economic and Social Research Council Methods Programme guidance on the conduct of narrative synthesis for systematic reviews (30). Reporting items of the synthesis of effect estimates without meta-analysis will follow the Synthesis Without Meta-Analysis (SWiM) guidelines (31).

We aim to synthesise results by distinct types of family forms (eg. single or coupled parents) and the age(s) at which a child is living in a single parent household. Where the age of the child during the exposure period or the duration of the exposure period is clearly reported, sub-group analyses will be carried out by age at which the child lived in a single parent household and by the length of exposure to single parenthood.

For each of the five main outcome groups, we will summarise the health and development outcomes most commonly reported and report any significant differences between children living with single parents and children living with coupled parents at any point during childhood. If any differences are reported between children living in different types of single parent households (separated single mothers vs never-married single mothers by choice), these will also be described. This will address our first research question. From included studies, we will identify factors such as employment or social support that influence differences in outcomes between children in single parent households and children in coupled parent households and may be responsive to policy change or intervention

For peer review only

targeting improvements in child health and development outcomes. This will address our second research question.

Should at least three studies employ the same design, and have similar exposure groups and outcomes, a decision will be made by the review team on whether meta-analysis is appropriate. A heterogeneity test (I^2 statistic) may be used to describe the percentage variability between studies and confirm whether it is reasonable to pool studies that appear comparable. Studies that have comparable exposures or outcomes but that are categorised as low quality based on the Newcastle-Ottawa scale however will not be included. Should a meta-analysis be appropriate, we will pool data using the DerSimonian and Laird random effects models (32). We will calculate odds ratios, presented using logarithmic scales will be calculated for dichotomous outcomes and weighted mean difference will be calculated for continuous outcomes. We will visualise results as forest plots.

Patient and public involvement

No patients were involved in the development of this protocol.

Ethics and Dissemination

No requests for ethical approval have been made given that all documents used are public accessible. We will submit results to a peer-reviewed journal for publication and international social science conferences. We will communicate results with single parent groups and relevant charitable organisations. This review will also be included in IL's thesis.

Authors' contributions

The protocol was conceived by all authors, written by IL in collaboration with PH and JW, and reviewed by AA and EI prior to submission.

Funding Statement

This work is supported by the NIHR Great Ormond Street Hospital Biomedical Research Centre. IL is funded by the Medical Research Council (grant reference MR/N013867/1).

Competiting Interests Statement

We declare no conflicts of interest.

For peer review only

1. Social Policy Division - Directorate of Employment LaSA. OECD Family Database - SF1.2 Children in Families 2020 [Available from: http://www.oecd.org/els/soc/SF_1_2_Children_in_families.pdf.
2. Statistics OoN. Families and households in the UK: 2019 2019 [Available from: https://www.ons.gov.uk/peoplepopulationandcommunity/birthsdeathsandmarriages/families/bulletins/familiesandhouseholds/2019.
3. Statistics OoN. Families and households in the UK: 2018 2019 [Available from: https://www.ons.gov.uk/peoplepopulationandcommunity/birthsdeathsandmarriages/families/bulletins/familiesandhouseholds/2018.
4. Rowlingson KMS. The Growth of Lone Parenthood: Diversity and Dynamics. London: Policy Studies Institute; 1998.
5. Bornstein MH. Being and Becoming a Parent: Single parenthood. Handbook of Parenting: Routledge; 2019.
6. Statistics OoN. Divorces in England and Wales: 2018. 2019.
7. Statistics OoN. Births by parents' characteristics 2019 [Available from: https://www.ons.gov.uk/peoplepopulationandcommunity/birthsdeathsandmarriages/livebirths/datasets/birthsbyparentscharacteristics.
8. Anderson J. The impact of family structure on the health of children: Effects of divorce. Linacre Q. 2014;81(4):378-87.
9. Amato PR. The Impact of Family Formation Change on the Cognitive, Social, and Emotional Well-Being of the Next Generation. Future Child. 2005;15(2):75-96.
10. Carlson MJ, Berger LM. What Kids Get from Parents: Packages of Parental Involvement across Complex Family Forms. Soc Serv Rev. 2013;87(2):213-49.
11. Weitoft GR, Hjern A, Haglund B, Rosén M. Mortality, severe morbidity, and injury in children living with single parents in Sweden: a population-based study. The Lancet. 2003;361(9354):289-95.
12. Lipman EL, Boyle MH, Dooley MD, Offord DR. Child Well-Being in Single-Mother Families. Journal of the American Academy of Child & Adolescent Psychiatry. 2002;41(1):75-82.
13. de Lange M, Dronkers J, Wolbers MHJ. Single-parent family forms and children's educational performance in a comparative perspective: effects of school's share of single-parent families. School Effectiveness and School Improvement. 2014;25(3):329-50.
14. Shah PS, Zao J, Ali S. Maternal Marital Status and Birth Outcomes: A Systematic Review and Meta-Analyses. Maternal and Child Health Journal. 2011;15(7):1097-109.
15. Duriancik DM, Goff CR. Children of single-parent households are at a higher risk of obesity: A systematic review. Journal of Child Health Care. 2019;23(3):358-69.
16. Rothwell DW, McEwen A. Comparing Child Poverty Risk by Family Structure During the 2008 Recession. Journal of Marriage and Family. 2017;79(5):1224-40.
17. Foundation JR. UK Poverty 2018: A comprehensive analysis of poverty trends and figures. 2018.
18. Nieuwenhuis R, Laurie CM. The triple bind of single-parent families. Bristol: Policy Press; 2018.
19. Rowlingson K, McKay S. Lone Motherhood and Socio-Economic Disadvantage: Insights from Quantitative and Qualitative Evidence. The Sociological Review. 2005;53(1):30-49.
20. Fomby P, Cherlin AJ. Family Instability and Child Well-Being. American Sociological Review. 2007;72(2):181-204.

For peer review only

21. Social Policy Division - Directorate of Employment LaSA. OECD Family database - SF1.1 Family size and household composition: OECD; 2016 [Available from: http://www.oecd.org/els/family/SF_1_1_Family_size_and_composition.pdf.
22. Lamb ME. The History of Research on Father Involvement. Marriage & Family Review. 2000;29(2-3):23-42.
23. Macfadyen A, Swallow V, Santacroce S, Lambert H. Involving fathers in research. Journal for Specialists in Pediatric Nursing. 2011;16(3):216-9.
24. Goldman RaB, Adrienne Wheres the daddy? Fathers and father—figures in UK datasets. 2017.
25. Afifi TO, Cox BJ, Enns MW. Mental health profiles among married, never-married, and separated/divorced mothers in a nationally representative sample. Social Psychiatry and Psychiatric Epidemiology. 2006;41(2):122-9.
26. Golombok S. Modern families: parents and children in new family forms: Cambridge : Cambridge University Press; 2015.
27. Carroll N. Lone mothers' experiences of stigma: A comparative study: Huddersfield; 2017.
28. Wells GS, B; O'Connell, D; Peterson, J; Welch, V; Losos, M; et al. Newcastle-Ottawa Scale (NOS) for assessing the quality of nonrandomized studies in meta-analyses. [Available from: http://www.ohri.ca/programs/clinical_epidemiology/oxford.asp.
29. Whiting P, Savović J, Higgins JPT, Caldwell DM, Reeves BC, Shea B, et al. ROBIS: A new tool to assess risk of bias in systematic reviews was developed. Journal of Clinical Epidemiology. 2016;69:225-34.
30. Popay J, Roberts H, Sowden A, Petticrew M, Arai L, Rodgers M, et al. Guidance on the conduct of narrative synthesis in systematic reviews: A product from the ESRC Methods Programme2006.
31. Campbell M, McKenzie JE, Sowden A, Katikireddi SV, Brennan SE, Ellis S, et al. Synthesis without meta-analysis (SWiM) in systematic reviews: reporting guideline. BMJ. 2020;368:l6890.
32. DG DJHJA. Analysing data and undertaking meta-analyses. 2019. In: Cochrane Handbook for Systematic Reviews of Interventions version 60 (updated July 2019) [Internet]. Cochrane. Available from: https://training.cochrane.org/handbook/current/chapter-10.

For peer review only

APPENDIX 1 – SEARCH STRATEGY

	**PUBMED**	
#1	single parents	"single parent"[MeSH Terms] OR "single parent*"[Title/Abstract] OR "lone parent*"[Title/Abstract] OR "lone mother*"[Title/Abstract] OR "lone father*"[Title/Abstract] OR "single father*"[Title/Abstract] OR "single mother*"[Title/Abstract] OR "single parent family"[Title/Abstract] OR "single parent families"[Title/Abstract] OR "single-parent family"[MeSH Terms] OR "unmarried parent*"[Title/Abstract] OR "marital status"[Title/Abstract] OR "marital status"[MeSH Terms] OR "family structure"[Title/Abstract]
#2	birth outcomes	"birth outcome*"[Title/Abstract] OR "birth weight"[MeSH Terms] OR "birth weight"[Title/Abstract] OR "infant, low birth weight"[MeSH Terms] OR "infant, premature"[MeSH Terms] OR "gestational age"[Title/Abstract] OR "preterm birth"[Title/Abstract] OR "congenital anomal*"[Title/Abstract]
#3	mortality	"child mortality"[Title/Abstract] OR "infant mortality"[Title/Abstract] OR "perinatal mortality"[Title/Abstract] OR "fetal mortality"[Title/Abstract] OR "stillbirth"[Title/Abstract] OR "stillbirth"[MeSH Terms]
#4	physical health	"child health"[MeSH Terms] OR "child health"[Title/Abstract] OR "pediatric obesity"[MeSH Terms] OR "child nutrition"[Title/Abstract] OR "eating habits"[Title/Abstract] OR "oral health"[Title/Abstract] OR "motor skills"[Title/Abstract]
#5	mental health and development	mental health[MeSH Terms] OR "educational status"[MeSH Terms] OR "cognitive abilit*"[Title/Abstract] OR "educational attainment"[Title/Abstract] OR "educational status"[Title/Abstract] OR "child behavior disorders"[MeSH Terms] OR "depressive disorder"[MeSH Terms] OR "anxiety disorders"[MeSH Terms]
#6	healthcare use	"hospital admission"[Title/Abstract] OR "emergency admission"[Title/Abstract] OR "healthcare use"[Title/Abstract] OR "health care use"[Title/Abstract] OR "healthcare utilisation"[Title/Abstract] OR "health visitor"[Title/Abstract] OR "primary care"[Title/Abstract] OR "vaccination"[MeSH Terms]
#7		#2 OR #3 OR #4 OR #5 OR #6
#8		#1 AND #7
#9		limit #8 to 2000-2020

	**SCOPUS**	
#1	single parents	TITLE-ABS ("single parent*" OR "lone parent*" OR "lone mother*" OR "lone father*" OR "single father*" OR "single mother*" OR "single parent family" OR "single parent families" OR "unmarried parent*" OR "marital status" OR "family structure")
#2	birth outcomes	TITLE-ABS ("birth outcome*" OR "birthweight" OR "gestational age" OR "preterm birth" OR "congenital anomal*")

For peer review only

#3	mortality	TITLE-ABS ("child mortality" OR "infant mortality" OR "perinatal mortality" OR "fetal mortality" OR "stillbirth")
#4	physical health	TITLE-ABS ("child health" OR "oral health" OR "pediatric obesity" OR "child nutrition" OR "eating habits" OR "motor skills")
#5	mental health and development	TITLE-ABS ("mental health" OR "disruptive behavior" OR "disruptive behaviour" OR "cognitive abilit*" OR "educational attainment" OR "educational status" OR "child development")
#6	healthcare use	TITLE-ABS ("healthcare use" OR "hospitalization" OR "hospitalisation" OR "hospital admission" OR "emergency admission" OR "vaccinat*")
#7		#2 OR #3 OR #4 OR #5 OR #6
#8		#1 AND #7
#9		limit #8 to 2000-2020

	**SCOPUS**	
#1	single parents	ti("single parent?" OR "lone parent?" OR "lone mother?" OR "lone father?" OR "single father?" OR "single mother?" OR "single parent family" OR "single parent families" OR "unmarried parent?" OR "marital status" OR "family structure") OR ab("single parent?" OR "lone parent?" OR "lone mother?" OR "lone father?" OR "single father?" OR "single mother?" OR "single parent family" OR "single parent families" OR "unmarried parent?" OR "marital status" OR "family structure")
#2	birth outcomes	ti("birth outcome?" OR "birthweight" OR "birth weight" OR "gestational age" OR "preterm birth" OR "congenital anomal?") OR ab("birth outcome?" OR "birthweight" OR "birth weight" OR "gestational age" OR "preterm birth" OR "congenital anomal?")
#3	mortality	ti("child mortality" OR "infant mortality" OR "perinatal mortality" OR "fetal mortality" OR "stillbirth") OR ab("child mortality" OR "infant mortality" OR "perinatal mortality" OR "fetal mortality" OR "stillbirth")
#4	physical health	ti("child health" OR "oral health" OR "pediatric obesity" OR "child nutrition" OR "eating habits" OR "motor skills") OR ab("child health" OR "oral health" OR "pediatric obesity" OR "child nutrition" OR "eating habits" OR "motor skills")
#5	mental health and development	ti("mental health" OR "disruptive behavior" OR "disruptive behaviour" OR "cognitive abilit?" OR "educational attainment" OR "educational status" OR "child development") OR ab("mental health" OR "disruptive behavior" OR "disruptive behaviour" OR "cognitive abilit?" OR "educational attainment" OR "educational status" OR "child development")
#6	healthcare use	ti("hospitalisation" OR "hospitalization" OR "hospital admission" OR "emergency admission" OR "healthcare use" OR "vaccinat?") OR ab("hospitalisation" OR "hospitalization" OR "hospital admission" OR "emergency admission" OR "healthcare use" OR "vaccinat?")

For peer review only

#7		#2 OR #3 OR #4 OR #5 OR #6
#8		#1 AND #7
#9		limit #8 to 2000-2020

For peer review only

PRISMA-P (Preferred Reporting Items for Systematic review and Meta-Analysis Protocols) 2015 checklist: recommended items to address in a systematic review protocol*

Section and topic	Item No	Checklist item	Page
**ADMINISTRATIVE INFORMATION**			
Title:			
Identification	1a	Identify the report as a protocol of a systematic review	1
Update	1b	If the protocol is for an update of a previous systematic review, identify as such	N/A
Registration	2	If registered, provide the name of the registry (such as PROSPERO) and registration number	2
Authors:			
Contact	3a	Provide name, institutional affiliation, e-mail address of all protocol authors; provide physical mailing address of corresponding author	1
Contributions	3b	Describe contributions of protocol authors and identify the guarantor of the review	7
Amendments	4	If the protocol represents an amendment of a previously completed or published protocol, identify as such and list changes; otherwise, state plan for documenting important protocol amendments	N/A
Support:			
Sources	5a	Indicate sources of financial or other support for the review	7
Sponsor	5b	Provide name for the review funder and/or sponsor	7
Role of sponsor or funder	5c	Describe roles of funder(s), sponsor(s), and/or institution(s), if any, in developing the protocol	N/A
**INTRODUCTION**			
Rationale	6	Describe the rationale for the review in the context of what is already known	3,4
Objectives	7	Provide an explicit statement of the question(s) the review will address with reference to participants, interventions, comparators, and outcomes (PICO)	5
**METHODS**			
Eligibility criteria	8	Specify the study characteristics (such as PICO, study design, setting, time frame) and report characteristics (such as years considered, language, publication status) to be used as criteria for eligibility for the review	5
Information sources	9	Describe all intended information sources (such as electronic databases, contact with study authors, trial registers or other grey literature sources) with planned dates of coverage	5
Search strategy	10	Present draft of search strategy to be used for at least one electronic database, including planned limits, such that it could be repeated	Appendix 1

For peer review only

Study records:			
Data management	11a	Describe the mechanism(s) that will be used to manage records and data throughout the review	6
Selection process	11b	State the process that will be used for selecting studies (such as two independent reviewers) through each phase of the review (that is, screening, eligibility and inclusion in meta-analysis)	6
Data collection process	11c	Describe planned method of extracting data from reports (such as piloting forms, done independently, in duplicate), any processes for obtaining and confirming data from investigators	6
Data items	12	List and define all variables for which data will be sought (such as PICO items, funding sources), any pre-planned data assumptions and simplifications	5-6
Outcomes and prioritization	13	List and define all outcomes for which data will be sought, including prioritization of main and additional outcomes, with rationale	5-6
Risk of bias in individual studies	14	Describe anticipated methods for assessing risk of bias of individual studies, including whether this will be done at the outcome or study level, or both; state how this information will be used in data synthesis	6-7
Data synthesis	15a	Describe criteria under which study data will be quantitatively synthesised	6-7
	15b	If data are appropriate for quantitative synthesis, describe planned summary measures, methods of handling data and methods of combining data from studies, including any planned exploration of consistency (such as I^2, Kendall's τ)	6-7
	15c	Describe any proposed additional analyses (such as sensitivity or subgroup analyses, meta-regression)	6-7
	15d	If quantitative synthesis is not appropriate, describe the type of summary planned	6-7
Meta-bias(es)	16	Specify any planned assessment of meta-bias(es) (such as publication bias across studies, selective reporting within studies)	6-7
Confidence in cumulative evidence	17	Describe how the strength of the body of evidence will be assessed (such as GRADE)	6-7

*** It is strongly recommended that this checklist be read in conjunction with the PRISMA-P Explanation and Elaboration (cite when available) for important clarification on the items. Amendments to a review protocol should be tracked and dated.** **The copyright for PRISMA-P (including checklist) is held by the PRISMA-P Group and is distributed under a Creative Commons Attribution Licence 4.0.**

From: Shamseer L, Moher D, Clarke M, Ghersi D, Liberati A, Petticrew M, Shekelle P, Stewart L, PRISMA-P Group. Preferred reporting items for systematic review and meta-analysis protocols (PRISMA-P) 2015: elaboration and explanation. BMJ. 2015 Jan 2;349(jan02 1):g7647.

For peer review only

Health outcomes, healthcare use and development in children born into or growing up in single-parent households: A systematic review study protocol

Journal:	*BMJ Open*
Manuscript ID	bmjopen-2020-043361.R1
Article Type:	Protocol
Date Submitted by the Author:	19-Dec-2020
Complete List of Authors:	Lut, Irina; UCL, GOS Institute of Child Health<br>Woodman, Jenny; UCL, Institute of Education<br>Armitage, Alice; UCL, GOS Institute of Child Health<br>Ingram, Elizabeth; UCL, Department of Applied Health Research<br>Harron, Katie; UCL Great Ormond Street Institute of Child Health Population Policy and Practice,<br>Hardelid, Pia; UCL, GOS Institute of Child Health
**Primary Subject Heading**:	Paediatrics
Secondary Subject Heading:	Public health
Keywords:	PAEDIATRICS, PUBLIC HEALTH, Community child health < PAEDIATRICS

SCHOLARONE™
Manuscripts

I, the Submitting Author has the right to grant and does grant on behalf of all authors of the Work (as defined in the below author licence), an exclusive licence and/or a non-exclusive licence for contributions from authors who are: i) UK Crown employees; ii) where BMJ has agreed a CC-BY licence shall apply, and/or iii) in accordance with the terms applicable for US Federal Government officers or employees acting as part of their official duties; on a worldwide, perpetual, irrevocable, royalty-free basis to BMJ Publishing Group Ltd ("BMJ") its licensees and where the relevant Journal is co-owned by BMJ to the co-owners of the Journal, to publish the Work in this journal and any other BMJ products and to exploit all rights, as set out in our licence.

The Submitting Author accepts and understands that any supply made under these terms is made by BMJ to the Submitting Author unless you are acting as an employee on behalf of your employer or a postgraduate student of an affiliated institution which is paying any applicable article publishing charge ("APC") for Open Access articles. Where the Submitting Author wishes to make the Work available on an Open Access basis (and intends to pay the relevant APC), the terms of reuse of such Open Access shall be governed by a Creative Commons licence – details of these licences and which Creative Commons licence will apply to this Work are set out in our licence referred to above.

Other than as permitted in any relevant BMJ Author's Self Archiving Policies, I confirm this Work has not been accepted for publication elsewhere, is not being considered for publication elsewhere and does not duplicate material already published. I confirm all authors consent to publication of this Work and authorise the granting of this licence.

For peer review only

Title

Health outcomes, healthcare use and development in children born into or growing up in single-parent households: A systematic review study protocol

Authors
Irina Lut[1], Jenny Woodman[2], Alice Armitage[1], Elizabeth Ingram[3], Katie Harron[1], Pia Hardelid[1]

1. UCL GOS Institute of Child Health
2. UCL Institute of Education
3. UCL Department of Applied Health Research

Email addresses: i.lut@ucl.ac.uk ; j.woodman@ucl.ac.uk ; alice.armitage.13@ucl.ac.uk ; e.ingram.17@ucl.ac.uk ; k.harron@ucl.ac.uk ; p.hardelid@ucl.ac.uk

Corresponding author:
Irina Lut
Population, Policy and Practice Research and Teaching Department
UCL GOS Institute of Child Health
30 Guildford St, London WC1N 1EH
Tel: 07960087974
Email: i.lut@ucl.ac.uk

Tables: 0
Figures: 0
Appendices: 1
Word Count: 3225

For peer review only

Abstract

Introduction

Up to a quarter of all children globally live in single-parent households. Studies have concluded that children who grow up with continuously married parents have better health outcomes than children who grow up with single or separated parents. This is consistent for key health and development outcomes including physical health, psychological wellbeing and educational attainment. Possible explanations include higher poverty and time limitations of parental engagement within single-parent families, but these only represent a narrow range of mechanisms. We aim to identify and synthesise the evidence on how being born into and/or living in a single-parent household compared to living in a two-parent household as a child impacts on health and development outcomes, healthcare use, and factors that may be driving differences.

Methods and Analysis

We will search Pubmed, Scopus, and ERIC and adapt our search terms for search engines and grey literature sites to include relevant conference abstracts and grey literature. We will restrict results to English language publications from 2000-2020 and screen against inclusion criteria. We will categorise main outcomes into five groups of outcomes: birth outcomes, mortality, physical health, mental health and development, and healthcare use. We will use the Newcastle-Ottawa scale to assess the methodological quality of studies. Narrative synthesis will form the primary analysis in the review. Synthesis of effect estimates without meta-analysis will follow the Synthesis Without Meta-Analysis (SWiM) guidelines.

Ethics and Dissemination

All documents used are publicly accessible. We will submit results to a peer-reviewed journal and international social science conferences. We will communicate results with single parent groups and relevant charitable organisations. This review will also be included in IL's PhD thesis.

Registration Details

The protocol has been accepted to the International Prospective Register for Systematic Reviews (PROSPERO) database, registration number CRD42020197890.

Article Summary

Strengths and limitations of this study:

- This review will fill an evidence gap on the drivers and protective factors that influence the health and development of children growing up in single-parent households
- A robust methodology and extensive search strategy will support clear results to inform policies and interventions to support single-parent households

For peer review only

- Findings from included studies will likely be heterogenous in terms of definitions of single-parent households, and definitions and measurements of outcomes, which may preclude meta-analysis

Introduction

Between 10% and 25% of children in member countries of the Organisation for Economic Cooperation and Development (OECD) live in single-parent households (1). In Great Britain, 15% of all families are headed by a single parent (2). The proportion of single-parent families in Great Britain has remained stable over the last 20 years (3) following an increase of single-parent families between 1970 and 1995 (4). The key reasons for this increase were rising levels of divorce and partnership breakdown during the 1970s and 1980s (2, 5) and an increase in the number of births to single women since the mid-1980s (4, 6, 7).

Multiple studies have concluded that children who grow up with continuously married parents have better outcomes than children who grow up with single parents or children whose parents separate during childhood (8-10). This is consistent for key health and development outcomes including physical health (11), psychological wellbeing (12) and educational attainment (13).

A systematic review of maternal marital status and birth outcomes from 2010 has summarised the current literature on risks of an infant being born with low birth weight (<2500 g), preterm birth (<37 weeks gestational age) or small for gestational age (below the 10th percentile for babies of the same gestational age) among married and unmarried women (14). Findings identify significantly increased odds of low birth weight, preterm birth and small for gestational age births among unmarried women compared to married women. A further systematic review found that children in single-parent households have higher body mass index and obesogenic behaviours such as insufficient physical activity and increased television viewing time, compared to children living with two parents (15).

Socio-economic factors such as income, occupation and education (also referred to as socio-economic status or SES), are strongly associated with both parental and child wellbeing (16). Children of single parents are at higher risk of living in poverty and deprivation compared to children growing up in coupled families (17). In 2018, 49% of children in single-parent families lived in poverty in England compared to 25% of children in coupled families (18). Women, who head approximately 88% of single-parent families globally (19), have lower earnings than men on average due to gender wage gaps and salary penalties for motherhood (20). Additionally, though the majority of single parents in OECD countries are in some form of paid employment, single parents are more likely to work in occupations with lower earning potential and job security, and must balance work responsibilities with childcare (21). While it is clear that single-parent families are disadvantaged with respect to socioeconomic status and health outcomes, it remains unclear whether SES fully explains differences in outcomes for children of single mothers compared to children with two parents. Other potential mechanisms linking single parenthood to a higher risk of adverse child health outcomes have been reported, including parenting stress, lack of social networks and support, and social stigma which can influence maternal mental health and effective parenting (11, 22). It is difficult to examine the extent to which each factor may

For peer review only

individually affect the association between living with a single parent and adverse health in children, particularly since the likelihood of becoming a single mother is very strongly associated with SES. Women with lower SES (based on their father's occupation) were up to six times more likely to become single mothers in a study of three large British surveys (23). The rate of relationship breakdown resulting in single motherhood was found to be almost double among women in unskilled work compared to women in professional or managerial roles (23).

A substantial body of research also exists on the health impact of parental relationship breakdown (a mode of entry into single parenthood) or reforming, on child health (24, 25). However, a review of literature up to 2005 concludes that children with two continuously married parents attain better cognitive and emotional outcomes compared to children with only one biological parent in the household (9) and this is more plausibly explained by higher deprivation and lower education among single mothers than by the impacts of a parental relationship breakdown (4).

Research on single parenthood has focused largely on single mothers. More often than men, women parent alone due to breakdown of a relationship or pursue parenthood without a partner from the point of conception (4). Research on the quality and quantity of fathering exists but has tended to focus on the impact of father absence rather than single-father families (26). There are well-described challenges to capturing fathers in research exploring the impact of parents on children's outcomes (27, 28); even less is known about different characteristics or sub-groups of single fathers than single mothers.

Historically, official statistics agencies have relied heavily on marital status to define single motherhood. The definition of a family, which has been based on blood or marriage ties in countries like the United States, drives the classification of one or two-parent families (29). Unmarried women who have had children with cohabiting partners and lived in a two-parent household have previously been grouped with single mothers, leading to inflation of the number of families that appeared to be led by non-partnered women (4). This is despite cohabiting households with children being one of the fastest growing family forms between 1980s-2000s in the UK and other countries (3). Single mothers who have separated from a partner, either via divorce or relationship breakdown, are likely to be different from women with no partner who become mothers (4) and report different parenting experiences (30). Capturing nuances in single-parent households may be critical in understanding why children of single mothers have poorer outcomes compared to children of coupled mothers and identifying protective factors. Distinguishing between different types of and all routes into single parenthood is important as family structures have become more complex and new non-traditional family forms are being recognised (4, 31). Definitions and terminology matter not only to make sure we understand the comparisons we are making between groups but also to ensure that negative or stigmatising narratives associated with single motherhood are not perpetuated (32).

In this systematic review we will compare a range of health and development outcomes among children living in single-parent households and children living in coupled-parent households, and identify factors that may be driving differences. We will focus particularly on children who remain in single-parent versus continuously coupled families but also

For peer review only

include comparisons with cohabiting and married coupled families, or sub-groups of single-parent families (separated single mothers, never-married single mothers by choice, single fathers) where available . We aim to fill gaps in evidence by exploring whether health disparities between children of single parents and children of coupled parents persist after taking into account socioeconomic characteristics, presenting findings that explain the differences and reporting protective factors that allow children to be healthy in a single-parent family. Our findings will highlight areas where policy change or public health interventions might help improve health of the large numbers of children living in single-parent households.

Methods and Analysis

Aims and research questions
The aim of this review is to systematically identify and synthesise the evidence on how being born into and/or living in a single-parent household as a child impacts on health outcomes, healthcare use and development outcomes, compared to living in a two-parent household, and factors that may be driving differences.

This systematic review will answer the following questions:

1. How do health, healthcare use and development outcomes compare among children and young people (less than 18 years old) growing up in single-parent and coupled-parent households?
2. What factors influence any observed differences in child health, healthcare use and development outcomes between children of single parents versus coupled parents?

Searches
We will search for the concepts 'single parents' AND 'child health' OR 'child development' outcomes using indexed medical subject headings (MeSH) and free text terms, restricting results to English language publications from 2000-2020. We will search three databases which index medical, social science and education research: Pubmed, Scopus, and ERIC. We will identify additional relevant results through backwards and forwards citation searching and grey literature search engines. We provide the full list of search concepts and terms in appendix 1 (carried out on 15.07.20). We will adapt our search terms for search engines like Google Scholar and Scirus, and refer to the Canadian Agency for Drugs and Technologies in Health (CADTH) guidance for links to grey literature sites relevant in the UK context, to search for conference abstracts and grey literature or additional peer-reviewed articles.

Two researchers (IL and AA or IL and EI) will independently screen all results based on title and abstract and further screen full texts for inclusion. A third reviewer (PH or JW) will resolve any discrepancies.

Inclusion and exclusion criteria
The population of interest is children who have experienced living in a single-parent household at any time during childhood (aged less than 18 years) and have at least one of the health outcomes measured in the study before the age of 18 years. We will include studies if the single parent (either mother or father) is living with dependent children and

For peer review only

does not have a partner living in the same home. Only studies with enough information to identify the single parent exposure group as we have defined it here will be included; studies where the exposure groups are married vs unmarried, without further specification of cohabitation status of parents, will be excluded.

Parents may transition in and out of relationships with different people (4, 33). While they may be consistently partnered, changes in family structure (also referred to as family instability) have also been shown to negatively impact on child outcomes (24). In this review, we will include studies that categorise children as 'ever having lived in a single-parent family during childhood' if the health impact of living with a single parent is also examined.

A substantial body of work shows that parental conflict and poor marital quality adversely affect behavioural outcomes, anxiety and depression and emotional security in children and adolescents (34, 35). However, in this systematic review we will exclude studies that focus exclusively on the health effects of parental relationship breakdown or quality without investigating the effects of single parenthood.

We will include studies with any definition and measure of the five types of outcomes. Studies employing quantitative study designs such as cohort, cross-sectional and case control studies will be included. A range of study types will provide a comprehensive view of the literature with a mix of well-powered studies, longitudinal data points and objectively measured outcomes.

Outcomes

We present the main outcomes in this review in five groups:

1. Birth outcomes: including birth weight, low birth weight (<2500g), very low birth weight (<1500g), gestational age, small for gestational age (<10th percentile), preterm birth, congenital anomalies
2. Mortality outcomes: including stillbirth, perinatal mortality, child mortality
3. Physical health outcomes: including nutrition, weight, oral health, motor skills
4. Mental health and development outcomes: including disruptive behaviour, substance abuse, anxiety or depressive disorders, autism-spectrum disorders, psychosis, self-harm and suicidality, cognitive abilities (problem solving, memory, language/communication, early years educational attainment), social-emotional development (personal-social skills)
5. Healthcare use outcomes: including any hospital admission (planned or emergency), vaccinations, visits to primary care, contact with health visitors or well-child checks

Data extraction and management

For each included study, we will extract information on study authors and date of publication, study setting (country and its World Bank income group classification if available) and period (year), study design (including selection criteria, number of participants and analysis, causal claims), timings and definition of single parenthood and

For peer review only - http://bmjopen.bmj.com/site/about/guidelines.xhtml

outcomes of the study. If available, we will additionally extract information about confounding variables that were controlled for, variables reported as effect modifiers of the relationship between single parenthood and child health and development, and variables that act as measures of socioeconomic status (for example, use of income support or tax credits, employment or access to health insurance) All management of included publications and extracted data will be done within the EPPI-Reviewer software.

Studies will be grouped by exposure groups (single-parent vs coupled-parent household) and main outcomes. Definitions of single parenthood may vary across studies and exposure groups are likely to differ by the type, timing and duration of parental relationship status. In most studies, the exposure is expected to be self-reported exposure or obtained from an administrative data source. Authors will be contacted if the time parameters of single parenthood are not clear from the published work.

Outcomes will be grouped into the five main outcome groups defined above. We will use the Newcastle-Ottawa scale to assess the methodological quality of studies (36). We will use the ROBINS-I tool for assessing risk of bias in non-randomised studies (37).

Synthesis and meta-analysis

Given the range of outcomes and the likely diversity in the way single-parent households are defined across studies, we expect that the included studies will be too heterogeneous to carry out meta-analyses. Narrative synthesis will therefore form the primary analysis in the review. To carry out a robust narrative synthesis, we will incorporate the four main elements described in the Economic and Social Research Council Methods Programme guidance on the conduct of narrative synthesis for systematic reviews (38). We will follow the nine reporting items of the synthesis of effect estimates without meta-analysis from the Synthesis Without Meta-Analysis (SWiM) guidelines (39) and discuss the limitations of the synthesis methods used.

We will first report how single parents are defined in each study, creating a typology based on the literature which will inform how studies are categorised for synthesis or subgroup meta-analysis. We then aim to synthesise results by distinct types of family forms (eg. single or coupled parents, married or cohabiting, single mother or single father) and the age(s) at which a child is living in a single-parent household. Where the age of the child during the exposure period or the duration of the exposure period is clearly reported, sub-group analyses will be carried out by age at which the child lived in a single parent household and by the length of exposure to single parenthood. Sub-group analyses will also be carried out separating single mothers and single fathers. Additional sub-group analyses or special attention in reporting will be considered to take into account socioeconomic status (based on SES indicators as available) and country context (based on World Bank income group classification) that could influence the association between single parents and child health.

For each of the five main outcome groups, we will summarise the health and development outcomes most commonly reported and report any significant differences between children living with single parents and children living with coupled parents at any point during childhood. If any differences are reported between children living in different sub-groups of single-parent households (separated single mothers vs never-married single mothers by

For peer review only

choice vs single fathers), these will also be described. This will address our first research question. From included studies, we will identify factors such as employment or social support that influence differences in outcomes between children in single-parent households and children in coupled-parent households. Identifying potential mechanisms impacting the relationship between single parenthood and child health (for example, access or family income) may inform policy change or intervention targeting improvements in child health and development outcomes. This will address our second research question.

Should at least three studies employ the same design, and have similar exposure groups and outcomes, a decision will be made by the review team on whether meta-analysis is appropriate. A heterogeneity test (I^2 statistic) may be used to describe the percentage variability between studies and confirm whether it is reasonable to pool studies that appear comparable. Studies that have comparable exposures or outcomes but that are categorised as low quality based on the Newcastle-Ottawa scale will not be included. Should a meta-analysis be appropriate, we will pool data using the DerSimonian and Laird random effects models (40). We will calculate adjusted measures of association (such as odds ratios, hazard rates and relative risk) presented using logarithmic scales, for dichotomous categorical outcomes and standardised mean difference for continuous outcomes. We will carry out separate meta-analyses for unadjusted and adjusted effect sizes to better understand the effects of confounding variables on the association between single parenthood and child outcomes. We will visualise results as forest plots. We will use funnel plots to assess publication bias (41).

Patient and public involvement

No patients were involved in the development of this protocol.

Ethics and Dissemination

No requests for ethical approval have been made given that all documents used are publicly accessible. We will submit results to a peer-reviewed journal for publication and international social science conferences. We will communicate results with single parent groups and relevant charitable organisations. This review will also be included in IL's PhD thesis.

Authors' contributions

The protocol was conceived by all authors, written by IL in collaboration with PH, KH and JW, and reviewed by AA and EI prior to submission.

Funding Statement

This work is supported by the NIHR Great Ormond Street Hospital Biomedical Research Centre (no award/grant number applicable). IL is funded by the Medical Research Council (grant reference MR/N013867/1).

Competing Interests Statement

We declare no conflicts of interest.

For peer review only

1. Social Policy Division - Directorate of Employment LaSA. OECD Family Database - SF1.2 Children in Families 2020 [Available from: http://www.oecd.org/els/soc/SF_1_2_Children_in_families.pdf.
2. Statistics OoN. Families and households in the UK: 2019 2019 [Available from: https://www.ons.gov.uk/peoplepopulationandcommunity/birthsdeathsandmarriages/families/bulletins/familiesandhouseholds/2019.
3. Statistics OoN. Families and households in the UK: 2018 2019 [Available from: https://www.ons.gov.uk/peoplepopulationandcommunity/birthsdeathsandmarriages/families/bulletins/familiesandhouseholds/2018.
4. Rowlingson KMS. The Growth of Lone Parenthood: Diversity and Dynamics. London: Policy Studies Institute; 1998.
5. Bornstein MH. Being and Becoming a Parent: Single parenthood. Handbook of Parenting: Routledge; 2019.
6. Statistics OoN. Divorces in England and Wales: 2018. 2019.
7. Statistics OoN. Births by parents' characteristics 2019 [Available from: https://www.ons.gov.uk/peoplepopulationandcommunity/birthsdeathsandmarriages/livebirths/datasets/birthsbyparentscharacteristics.
8. Anderson J. The impact of family structure on the health of children: Effects of divorce. Linacre Q. 2014;81(4):378-87.
9. Amato PR. The Impact of Family Formation Change on the Cognitive, Social, and Emotional Well-Being of the Next Generation. Future Child. 2005;15(2):75-96.
10. Carlson MJ, Berger LM. What Kids Get from Parents: Packages of Parental Involvement across Complex Family Forms. Soc Serv Rev. 2013;87(2):213-49.
11. Weitoft GR, Hjern A, Haglund B, Rosén M. Mortality, severe morbidity, and injury in children living with single parents in Sweden: a population-based study. The Lancet. 2003;361(9354):289-95.
12. Lipman EL, Boyle MH, Dooley MD, Offord DR. Child Well-Being in Single-Mother Families. Journal of the American Academy of Child & Adolescent Psychiatry. 2002;41(1):75-82.
13. de Lange M, Dronkers J, Wolbers MHJ. Single-parent family forms and children's educational performance in a comparative perspective: effects of school's share of single-parent families. School Effectiveness and School Improvement. 2014;25(3):329-50.
14. Shah PS, Zao J, Ali S. Maternal Marital Status and Birth Outcomes: A Systematic Review and Meta-Analyses. Maternal and Child Health Journal. 2011;15(7):1097-109.
15. Duriancik DM, Goff CR. Children of single-parent households are at a higher risk of obesity: A systematic review. Journal of Child Health Care. 2019;23(3):358-69.
16. Nieuwenhuis R, Laurie CM. The triple bind of single-parent families. Bristol: Policy Press; 2018.
17. Rothwell DW, McEwen A. Comparing Child Poverty Risk by Family Structure During the 2008 Recession. Journal of Marriage and Family. 2017;79(5):1224-40.
18. Foundation JR. UK Poverty 2018: A comprehensive analysis of poverty trends and figures. 2018.
19. Social Policy Division - Directorate of Employment LaSA. OECD Family database - SF1.1 Family size and household composition: OECD; 2016 [Available from: http://www.oecd.org/els/family/SF_1_1_Family_size_and_composition.pdf.

For peer review only - http://bmjopen.bmj.com/site/about/guidelines.xhtml

20. Budig MJ, Misra J, Boeckmann I. Work–Family Policy Trade-Offs for Mothers? Unpacking the Cross-National Variation in Motherhood Earnings Penalties. Work and Occupations. 2015;43(2):119-77.
21. Maldonado RNaLC. Single-parent families and in-work poverty. In: Marx HLaI, editor. Handbook on In-Work Poverty. Cheltenham, UK: Edward Elgar Publishing; 2018. p. 171-92.
22. Weinraub M, Wolf BM. Effects of stress and social supports on mother-child interactions in single- and two-parent families. Child development. 1983;54(5):1297-311.
23. Rowlingson K, McKay S. Lone Motherhood and Socio-Economic Disadvantage: Insights from Quantitative and Qualitative Evidence. The Sociological Review. 2005;53(1):30-49.
24. Fomby P, Cherlin AJ. Family Instability and Child Well-Being. American Sociological Review. 2007;72(2):181-204.
25. Amato PR. Research on Divorce: Continuing Trends and New Developments. Journal of Marriage and Family. 2010;72(3):650-66.
26. Lamb ME. The History of Research on Father Involvement. Marriage & Family Review. 2000;29(2-3):23-42.
27. Macfadyen A, Swallow V, Santacroce S, Lambert H. Involving fathers in research. Journal for Specialists in Pediatric Nursing. 2011;16(3):216-9.
28. Goldman RaB, Adrienne Wheres the daddy? Fathers and father—figures in UK datasets. 2017.
29. Bianchi SM. The Changing Demographic and Socioeconomic Characteristics of Single Parent Families. Marriage & Family Review. 1994;20(1-2):71-97.
30. Afifi TO, Cox BJ, Enns MW. Mental health profiles among married, never-married, and separated/divorced mothers in a nationally representative sample. Social Psychiatry and Psychiatric Epidemiology. 2006;41(2):122-9.
31. Golombok S. Modern families: parents and children in new family forms: Cambridge : Cambridge University Press; 2015.
32. Carroll N. Lone mothers' experiences of stigma: A comparative study: Huddersfield; 2017.
33. Beck AN, Cooper CE, McLanahan S, Brooks-Gunn J. Partnership Transitions and Maternal Parenting. Journal of marriage and the family. 2010;72(2):219-33.
34. Hair E, Moore K, Hadley A, Kaye K, Day R, Orthner D. Parent Marital Quality and the Parent–Adolescent Relationship: Effects on Adolescent and Young Adult Health Outcomes. Marriage and Family Review - MARRIAGE FAM REV. 2009;45:218-48.
35. Vaez E, Indran R, Abdollahi A, Juhari R, Mansor M. How marital relations affect child behavior: review of recent research. Vulnerable Children and Youth Studies. 2015;10(4):321-36.
36. Wells GS, B; O'Connell, D; Peterson, J; Welch, V; Losos, M; et al. Newcastle-Ottawa Scale (NOS) for assessing the quality of nonrandomized studies in meta-analyses. [Available from: http://www.ohri.ca/programs/clinical_epidemiology/oxford.asp.
37. Sterne JAC, Hernán MA, Reeves BC, Savović J, Berkman ND, Viswanathan M, et al. ROBINS-I: a tool for assessing risk of bias in non-randomised studies of interventions. BMJ. 2016;355:i4919.
38. Popay J, Roberts H, Sowden A, Petticrew M, Arai L, Rodgers M, et al. Guidance on the conduct of narrative synthesis in systematic reviews: A product from the ESRC Methods Programme2006.

For peer review only

39. Campbell M, McKenzie JE, Sowden A, Katikireddi SV, Brennan SE, Ellis S, et al. Synthesis without meta-analysis (SWiM) in systematic reviews: reporting guideline. BMJ. 2020;368:l6890.
40. DG DJHJA. Analysing data and undertaking meta-analyses. 2019. In: Cochrane Handbook for Systematic Reviews of Interventions version 60 (updated July 2019) [Internet]. Cochrane. Available from: https://training.cochrane.org/handbook/current/chapter-10.
41. Sterne JA, Egger M, Smith GD. Systematic reviews in health care: Investigating and dealing with publication and other biases in meta-analysis. BMJ. 2001;323(7304):101-5.

For peer review only

APPENDIX 1 – SEARCH STRATEGY

	PUBMED	
#1	single parents	"single parent"[MeSH Terms] OR "single parent*"[Title/Abstract] OR "lone parent*"[Title/Abstract] OR "lone mother*"[Title/Abstract] OR "lone father*"[Title/Abstract] OR "single father*"[Title/Abstract] OR "single mother*"[Title/Abstract] OR "single parent family"[Title/Abstract] OR "single parent families"[Title/Abstract] OR "single-parent family"[MeSH Terms] OR "unmarried parent*"[Title/Abstract] OR "marital status"[Title/Abstract] OR "marital status"[MeSH Terms] OR "family structure"[Title/Abstract]
#2	birth outcomes	"birth outcome*"[Title/Abstract] OR "birth weight"[MeSH Terms] OR "birth weight"[Title/Abstract] OR "infant, low birth weight"[MeSH Terms] OR "infant, premature"[MeSH Terms] OR "gestational age"[Title/Abstract] OR "preterm birth"[Title/Abstract] OR "congenital anomal*"[Title/Abstract]
#3	mortality	"child mortality"[Title/Abstract] OR "infant mortality"[Title/Abstract] OR "perinatal mortality"[Title/Abstract] OR "fetal mortality"[Title/Abstract] OR "stillbirth"[Title/Abstract] OR "stillbirth"[MeSH Terms]
#4	physical health	"child health"[MeSH Terms] OR "child health"[Title/Abstract] OR "pediatric obesity"[MeSH Terms] OR "child nutrition"[Title/Abstract] OR "eating habits"[Title/Abstract] OR "oral health"[Title/Abstract] OR "motor skills"[Title/Abstract]
#5	mental health and development	mental health[MeSH Terms] OR "educational status"[MeSH Terms] OR "cognitive abilit*"[Title/Abstract] OR "educational attainment"[Title/Abstract] OR "educational status"[Title/Abstract] OR "child behavior disorders"[MeSH Terms] OR "depressive disorder"[MeSH Terms] OR "anxiety disorders"[MeSH Terms]
#6	healthcare use	"hospital admission"[Title/Abstract] OR "emergency admission"[Title/Abstract] OR "healthcare use"[Title/Abstract] OR "health care use"[Title/Abstract] OR "healthcare utilisation"[Title/Abstract] OR "health visitor"[Title/Abstract] OR "primary care"[Title/Abstract] OR "vaccination"[MeSH Terms]
#7		#2 OR #3 OR #4 OR #5 OR #6
#8		#1 AND #7
#9		limit #8 to 2000-2020

	SCOPUS	
#1	single parents	TITLE-ABS ("single parent*" OR "lone parent*" OR "lone mother*" OR "lone father*" OR "single father*" OR "single mother*" OR "single parent family" OR "single parent families" OR "unmarried parent*" OR "marital status" OR "family structure")
#2	birth outcomes	TITLE-ABS ("birth outcome*" OR "birthweight" OR "gestational age" OR "preterm birth" OR "congenital anomal*")
#3	mortality	TITLE-ABS ("child mortality" OR "infant mortality" OR "perinatal mortality" OR "fetal mortality" OR "stillbirth")

For peer review only

#4	physical health	TITLE-ABS ("child health" OR "oral health" OR "pediatric obesity" OR "child nutrition" OR "eating habits" OR "motor skills")
#5	mental health and development	TITLE-ABS ("mental health" OR "disruptive behavior" OR "disruptive behaviour" OR "cognitive abilit*" OR "educational attainment" OR "educational status" OR "child development")
#6	healthcare use	TITLE-ABS ("healthcare use" OR "hospitalization" OR "hospitalisation" OR "hospital admission" OR "emergency admission" OR "vaccinat*")
#7		#2 OR #3 OR #4 OR #5 OR #6
#8		#1 AND #7
#9		limit #8 to 2000-2020

	**SCOPUS**	
#1	single parents	ti("single parent?" OR "lone parent?" OR "lone mother?" OR "lone father?" OR "single father?" OR "single mother?" OR "single parent family" OR "single parent families" OR "unmarried parent?" OR "marital status" OR "family structure") OR ab("single parent?" OR "lone parent?" OR "lone mother?" OR "lone father?" OR "single father?" OR "single mother?" OR "single parent family" OR "single parent families" OR "unmarried parent?" OR "marital status" OR "family structure")
#2	birth outcomes	ti("birth outcome?" OR "birthweight" OR "birth weight" OR "gestational age" OR "preterm birth" OR "congenital anomal?") OR ab("birth outcome?" OR "birthweight" OR "birth weight" OR "gestational age" OR "preterm birth" OR "congenital anomal?")
#3	mortality	ti("child mortality" OR "infant mortality" OR "perinatal mortality" OR "fetal mortality" OR "stillbirth") OR ab("child mortality" OR "infant mortality" OR "perinatal mortality" OR "fetal mortality" OR "stillbirth")
#4	physical health	ti("child health" OR "oral health" OR "pediatric obesity" OR "child nutrition" OR "eating habits" OR "motor skills") OR ab("child health" OR "oral health" OR "pediatric obesity" OR "child nutrition" OR "eating habits" OR "motor skills")
#5	mental health and development	ti("mental health" OR "disruptive behavior" OR "disruptive behaviour" OR "cognitive abilit?" OR "educational attainment" OR "educational status" OR "child development") OR ab("mental health" OR "disruptive behavior" OR "disruptive behaviour" OR "cognitive abilit?" OR "educational attainment" OR "educational status" OR "child development")
#6	healthcare use	ti("hospitalisation" OR "hospitalization" OR "hospital admission" OR "emergency admission" OR "healthcare use" OR "vaccinat?") OR ab("hospitalisation" OR "hospitalization" OR "hospital admission" OR "emergency admission" OR "healthcare use" OR "vaccinat?")
#7		#2 OR #3 OR #4 OR #5 OR #6
#8		#1 AND #7
#9		limit #8 to 2000-2020

For peer review only

PRISMA-P (Preferred Reporting Items for Systematic review and Meta-Analysis Protocols) 2015 checklist: recommended items to address in a systematic review protocol*

Section and topic	Item No	Checklist item	Page
**ADMINISTRATIVE INFORMATION**			
Title:			
Identification	1a	Identify the report as a protocol of a systematic review	1
Update	1b	If the protocol is for an update of a previous systematic review, identify as such	N/A
Registration	2	If registered, provide the name of the registry (such as PROSPERO) and registration number	2
Authors:			
Contact	3a	Provide name, institutional affiliation, e-mail address of all protocol authors; provide physical mailing address of corresponding author	1
Contributions	3b	Describe contributions of protocol authors and identify the guarantor of the review	7
Amendments	4	If the protocol represents an amendment of a previously completed or published protocol, identify as such and list changes; otherwise, state plan for documenting important protocol amendments	N/A
Support:			
Sources	5a	Indicate sources of financial or other support for the review	7
Sponsor	5b	Provide name for the review funder and/or sponsor	7
Role of sponsor or funder	5c	Describe roles of funder(s), sponsor(s), and/or institution(s), if any, in developing the protocol	N/A
**INTRODUCTION**			
Rationale	6	Describe the rationale for the review in the context of what is already known	3,4
Objectives	7	Provide an explicit statement of the question(s) the review will address with reference to participants, interventions, comparators, and outcomes (PICO)	5
**METHODS**			
Eligibility criteria	8	Specify the study characteristics (such as PICO, study design, setting, time frame) and report characteristics (such as years considered, language, publication status) to be used as criteria for eligibility for the review	5
Information sources	9	Describe all intended information sources (such as electronic databases, contact with study authors, trial registers or other grey literature sources) with planned dates of coverage	5
Search strategy	10	Present draft of search strategy to be used for at least one electronic database, including planned limits, such that it could be repeated	Appendix 1

For peer review only

Study records:			
Data management	11a	Describe the mechanism(s) that will be used to manage records and data throughout the review	6
Selection process	11b	State the process that will be used for selecting studies (such as two independent reviewers) through each phase of the review (that is, screening, eligibility and inclusion in meta-analysis)	6
Data collection process	11c	Describe planned method of extracting data from reports (such as piloting forms, done independently, in duplicate), any processes for obtaining and confirming data from investigators	6
Data items	12	List and define all variables for which data will be sought (such as PICO items, funding sources), any pre-planned data assumptions and simplifications	5-6
Outcomes and prioritization	13	List and define all outcomes for which data will be sought, including prioritization of main and additional outcomes, with rationale	5-6
Risk of bias in individual studies	14	Describe anticipated methods for assessing risk of bias of individual studies, including whether this will be done at the outcome or study level, or both; state how this information will be used in data synthesis	6-7
Data synthesis	15a	Describe criteria under which study data will be quantitatively synthesised	6-7
	15b	If data are appropriate for quantitative synthesis, describe planned summary measures, methods of handling data and methods of combining data from studies, including any planned exploration of consistency (such as I^2, Kendall's τ)	6-7
	15c	Describe any proposed additional analyses (such as sensitivity or subgroup analyses, meta-regression)	6-7
	15d	If quantitative synthesis is not appropriate, describe the type of summary planned	6-7
Meta-bias(es)	16	Specify any planned assessment of meta-bias(es) (such as publication bias across studies, selective reporting within studies)	6-7
Confidence in cumulative evidence	17	Describe how the strength of the body of evidence will be assessed (such as GRADE)	6-7

*** It is strongly recommended that this checklist be read in conjunction with the PRISMA-P Explanation and Elaboration (cite when available) for important clarification on the items. Amendments to a review protocol should be tracked and dated.** **The copyright for PRISMA-P (including checklist) is held by the PRISMA-P Group and is distributed under a Creative Commons Attribution Licence 4.0.**

From: Shamseer L, Moher D, Clarke M, Ghersi D, Liberati A, Petticrew M, Shekelle P, Stewart L, PRISMA-P Group. Preferred reporting items for systematic review and meta-analysis protocols (PRISMA-P) 2015: elaboration and explanation. BMJ. 2015 Jan 2;349(jan02 1):g7647.

For peer review only
